# Effects of SFRP4 overexpression on the production of adipokines in transgenic mice

**DOI:** 10.1080/21623945.2020.1792614

**Published:** 2020-07-13

**Authors:** Yali Zhang, Hua Guan, Yu Fu, Xin Wang, Liang Bai, Sihai Zhao, Enqi Liu

**Affiliations:** a Research Institute of Atherosclerotic Disease, Xi’an Jiaotong University Cardiovascular Research Centre, Xi’an, Shaanxi, China; b Laboratory Animal Center, Xi’an Jiaotong University Health Science Centre, Xi’an, Shaanxi, China; c Shaanxi Key Laboratory of Ischemic Cardiovascular Diseases & Institute of Basic and Translational Medicine, Xi’an Medical University, Xi’an, ShaanXi, China

**Keywords:** Secreted frizzled-related protein 4, transgenic mice, adipokines, visceral adipose tissue, white adipose tissue

## Abstract

Secreted frizzled-related protein (SFRP) 4 is an extracellular antagonist of Wnt signalling that regulates adipogenesis, and is highly in the visceral adipose tissue of obese individuals. However, it is still unclear how exactly SFRP4 regulates the secretion of adipokines in the adipose tissue *in vivo*, an event that is closely related to the pathogenesis of obesity and insulin resistance. In this study, we generated transgenic (Tg) mice overexpressing *SFRP4* in the liver and investigated SFRP4 role in adipokine secretion in mice on a regular normal diet. In Tg mice, SFRP4 protein was overexpressed in the liver, as compared to wild-type littermates (non-Tg), and released into the blood. Moreover, the size of adipocytes was smaller in the visceral adipose tissue of Tg mice compared to controls. Additionally, *SFRP4* overexpression affected the expression of genes related to adipocyte differentiation, causing the upregulation of adiponectin and glucose transporter 4, and the downregulation of CCAAT/enhancer-binding protein-β, in both visceral and subcutaneous adipose tissue. However, there was no difference in body weight or body composition between Tg and non-Tg mice. In summary, our data showed that *SFRP4* overexpression altered adipocyte size and adipokine secretion, possibly affecting adipocyte differentiation, obesity, and glucose metabolism.

## Introduction

Obesity is a health risk factor, closely associated with chronic non-communicable diseases such as metabolic syndrome, type 2 diabetes, and hypertension [1]. With the increase in obesity rate, the incidence of type 2 diabetes, impaired glucose tolerance, and other obesity-related complications has escalated [2–4]. The adipose tissue secretes a wide variety of adipokines that participate in various physiological processes, including food intake, insulin sensitivity, immunity, and inflammation [5–7].

Secreted frizzled-related protein 4 (SFRP4) belongs to a family of secreted proteins containing a frizzled-like cysteine-rich domain, which binds to Wnt ligands or frizzled receptors extracellularly, thus modulating the Wnt cascade [8,9]. Wnt family proteins regulate adipocyte differentiation and lipid metabolism by paracrine or autocrine mechanisms [10–12]. SFRP4 is expressed in adipocytes, as well as in various tissues including pancreas, heart, bone, and uterus, and is involved in multiple pathophysiological processes including fibrosis, angiogenesis, apoptosis, malignancy, adipogenic differentiation, and bone metabolism [13–19]. In recent years, SFRP4 was reported to play a critical role in glucose and lipid metabolism [20,21]. First, SFRP4 is overexpressed in obese individuals and patients with type 2 diabetes [22,23]. Second, circulating SFRP4 significantly correlates with body fat percentage, body mass index, and insulin sensitivity [23]. Third, SFRP4 is overexpressed in the islets of patients with type 2 diabetes and is considered a marker of early beta cell dysfunction [24]. In addition, SFRP4 promotes the differentiation of human adipose tissue-derived mesenchymal stem cells into mature adipocytes [19].

Our previous study reported that *SFRP4* knockdown inhibits the differentiation of preadipocytes derived from visceral adipose tissue, while promoting the differentiation of precursor cells isolated from subcutaneous adipose tissue, indicating that SFRP4 may have distinct functions in different adipose depots [25]. In addition, we discovered that *SFRP4* knockdown in adipocytes downregulates the expression of adipokines, including adiponectin and glucose transporter 4 (GLUT4) [25]. In order to investigate the effect of SFRP4 on the expression of adipokines from adipose tissue *in vivo*, we generated transgenic mice with liver-specific *SFRP4* overexpression to increase the level of circulating SFRP4. Our results indicated that *SFRP4* overexpression affected the expression of adipokines, as well as the size of adipocytes.

## Materials and methods

### Animals

**Figure 1.**
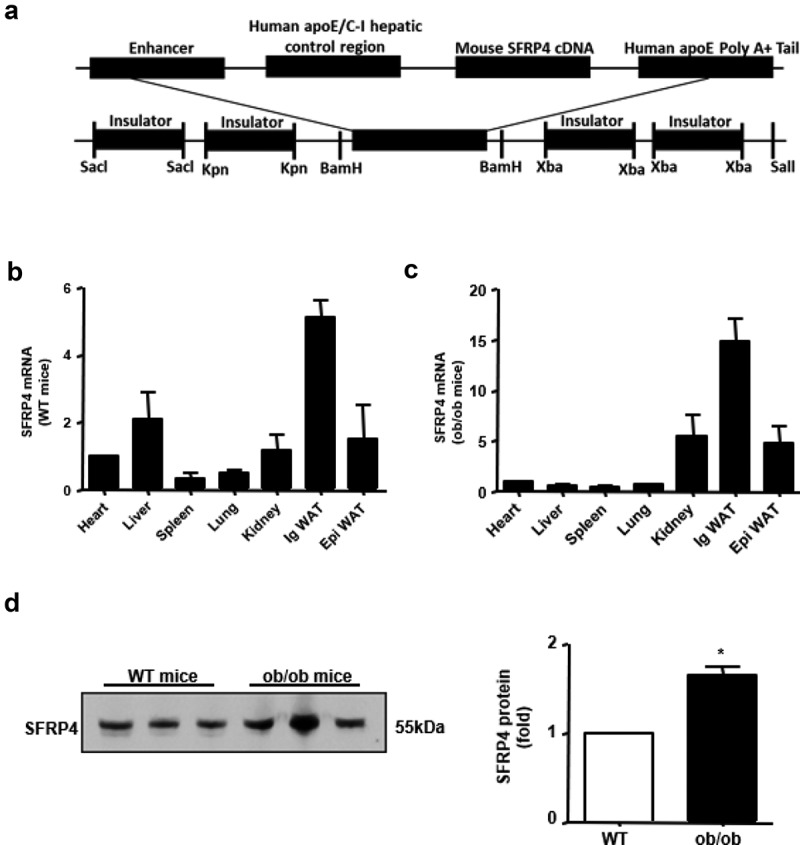
SFRP4 is highly expressed in ob/ob mice. (a) Construction of transgenic expression vector. (b) Expression of *SFRP4* in various tissues of wild type (WT) mice, as determined by qPCR. (c) Expression of *SFRP4* in various tissues of ob/ob mice, as determined by qPCR. (d) Western blotting analysis of SFRP4 expression in the plasma of WT and ob/ob mice. The data are expressed as the mean ± SEM, n = 6 for each group. *P < 0.05, vs. WT mice. ig WAT, inguinal white adipose tissue (subcutaneous adipose tissue); epi WAT, epididymal white adipose tissue (visceral adipose tissue)

Transgenic (Tg) mice of C57BL/6 N background were generated in our laboratory as previously reported [26,27]. The DNA construct used for microinjection contained mouse *SFRP4* cDNA under the control of the liver-specific sequences containing partial apoE and liver-specific region of the apoE/apoCI gene locus [28], along with four copies of the chicken β globin insulator (Figure 1(a)), to prevent position effects of transgene insulators [29]. The transgene was confirmed by PCR. F1 descendants from three of the five founders carried the gene insert, while the other two were chimeras. One of the three Tg founders, exhibiting the highest *SFRP4* mRNA expression level, was selected for progeny generation. Leptin-null (ob/ob) mice of C57BL/6 N background were purchased from Vital River Laboratory (Beijing Vital River Laboratory Animal Technologies Co. Ltd, Beijing, China). All mice were maintained in 12-h light/dark cycle, fed a standard rodent diet, and provided with water *ad libitum*. All animal procedures were performed in accordance with the guidelines of the Animal Care and Use Committee of Xi’an Jiaotong University.

### DNA extraction

About 0.5 cm of mouse tail was cut, then placed in a mixture of proteinase K and lysis buffer, digested overnight at 55°C, extracted with an organic solvent (saturated phenol: chloroform: isoamyl alcohol = 25:24:1), washed with 75% absolute ethanol, and finally dissolved in double-distilled water. DNA purity and concentration were measured by Nano Drop 2000 (ThermoFisher, Waltham, MA, USA).

### Polymerase chain reaction (PCR)

Genomic DNA samples were subjected to PCR analysis using a pair of primers specific for Tg mice (forward: 5ʹ- CCTGGCAACATACCTGAGCA-3ʹ and reverse: 5ʹ- CACTCCTCTGGACGGCTTTT-3ʹ). The 20-µL reactions included 8.2 µL of ddH_2_O, 10 µL of Taq DNA Polymerase mix, 1 µL of genomic DNA sample, 0.4 µL of 25 µmol/L Forward primer, and 0.4 µL of 25 µmol/L Reverse primer. The PCR conditions were as follows: 95°C 3 min (1 X), 94°C 30 s, 55°C 30 min, 72°C 45 s, 30 cycles, 72°C 10 min, 4°C 10 min. Equal amounts of PCR products were analysed by 1% agarose gels.

### RNA extraction and quantitative real-time PCR (qPCR)

Total RNA of adipose tissues was extracted by using RNAiso Plus (TaKaRa Biology Inc., Shiga, Japan). Reverse transcription was performed from 1 µg of total RNA by using the PrimeScript RT reagent kit (TaKaRa Biology Inc., Shiga, Japan). As we previously described [30], qPCR analysis was performed in triplicates, and the values were normalized to β-actin. Each 20-µL PCR reaction included 0.8 µL (10 µM) of forward and reverse primers and 10 µL of 2× SYBR Green PCR Master Mix. The PCR reactions were performed by using a Thermal Cycler Dice Real Time System TP-800 (TaKaRa Biology Inc., Shiga, Japan). The generation of specific PCR products was confirmed by melting curve analysis and gene expression levels were calculated by the comparative Ct method, X = 2^−ΔΔCt^. The primer sequences are shown in Table 1.

**Table 1.** The sequences of primers used in this study

Gene	Accession number		Sequence (5ʹ-3ʹ)
Adiponectin	NM_009605	Reverse	TTCTGTCTGTACGATTGTCAGTGG
		Forward	GTCATCTTCGGCATGACTGG
β-actin	NM_007393	Reverse	GCGGCATCCACGAAACTAC
		Forward	TGATCTCCTTCTGCATCCTGTC
C/EBPβ	NM_001287739	Reverse	ACCGGGTTTCGGGACTTGA
		Forward	CCCGCAGGAACATCTTTAAGTGA
PPARγ	NM_001127330	Reverse	GGAGCCTAAGTTTGAGTTTGCTGTG
		Forward	TGCAGCAGGTTGTCTTGGATG
SFRP4	NM_016687	Reverse	AAGCCGACCCTGGCAACATA
		Forward	TTGTGACCTCATTGCAACCACTC
PPARα	NM_001113418.1	Reverse	CAGAGCGCTAAGCTGTGATG
		Forward	CCCTGTGAACTGACGTTTGTG
Perilipin 2	NM_007408	Reverse	GATTGAATTCGCCAGGAAGA
		Forward	TGGCATGTAGTCTGGAGCTG
GLUT4	NM_001359114.1	Reverse	CCCATCCTTACGTCAGAGCC
		Forward	GCCCGGACCCTATACCCTAT

### Protein extraction and western blotting analysis

The adipose tissue and liver of mice were homogenized in ice-cold lysis buffer containing 1% protease inhibitors and phosphorylase inhibitors, then clarified by centrifugation (12, 000 g, 5 min). The protein content was determined by using a BCA protein concentration assay kit (Thermo Fisher, Waltham, MA, USA). After protein denaturation, the expression of target protein was evaluated by western blotting. Briefly, 30 µg of protein sample was subjected to 10% SDS-PAGE, transferred to polyvinylidene fluoride membranes (Bio-Rad, Hercules, CA, USA), and immunoblotted by using primary antibodies (Abs) against SFRP4 (1:1000; Cat. No.15328, Proteintech), adiponectin (1:1000; Cat. No.AF1119, R&D), fatty acid binding protein 4 (FABP4) (1:1000; Cat. No. AF1443, R&D), β-Actin (1:1000; Cat.No.SC69879, Santa Cruz) or β-Tubulin (1:2000; Cat. No. ab15246, Abcam) at 4°C overnight, as per the manufacturer’s instructions. After washing with tris-buffered saline-Tween-20, the membrane was incubated with horseradish peroxidase-conjugated secondary antibodies at room temperature (20°C – 25°C) for 1 h. After washing with tris-buffered saline, the antigen-antibody complexes were visualized by using an enhanced chemiluminescence detection kit (Millipore, Billerica, MA, USA). β-Actin or β-Tubulin was used as a loading control.

### Morphological analysis of adipose tissue

Adipose tissues were excised and fixed in 10% formalin buffer. The fixed specimens were processed to paraffin blocks, sectioned, and stained with haematoxylin-eosin (H&E) according to standard procedures [31]. The sections for microscopic quantification were photographed under a microscope equipped with a digital camera (Nikon, Tokyo, Japan). The diameter and area of adipocytes were measured by using Image J software (https://imagej.nih.gov/ij/).

### Statistical analysis

All data were expressed as the mean ± SEM. For statistical analyses, the Student’s t-test or the Welch’s t test were applied in case of equal or non-equal F values, respectively. *P* values < 0.05 were considered statistically significant. All analyses were performed by using GraphPad PRISM 5.0 (GraphPad Software, Inc, San Diego, CA, USA).

## Results

### SFRP4 was highly expressed in ob/ob mice

To determine the tissue distribution of SFRP4 in wild-type (WT) and ob/ob mice, we isolated the heart, liver, spleen, lung, kidney, inguinal white adipose tissue (ig WAT), and epididymal white adipose tissue (epi WAT) from WT and ob/ob mice to analyse SFRP4 mRNA expression. The results showed that in WT mice, SFRP4 expression was highest in the ig WAT, followed by the liver and epi WAT (Figure 1(b)). Similarly, in ob/ob mice, SFRP4 showed the highest expression in ig WAT, followed by kidney and epi WAT (Figure 1(c)). Plasma was collected from the tail vein and western blotting analysis was performed to measure SFRP4 protein expression. The results showed that the plasma level of SFRP4 protein was significantly higher in ob/ob mice than in WT mice (*P* < 0.05) (Figure 1(d)). The observed differences in SFRP4 expression between visceral and subcutaneous adipose tissue, as well as in plasma SFRP4 level between WT and ob/ob mice, suggested that SFRP4 played an important role in adipogenesis and adipocyte differentiation.

### The expression of SFRP4 was increased in Tg mice

**Figure 2.**
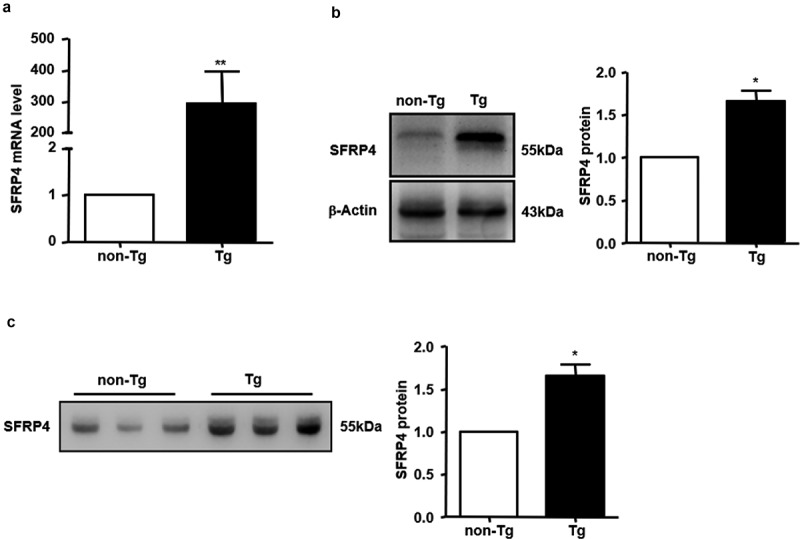
The expression of *SFRP4* was increased in Tg mice. (a) *SFRP4* mRNA expression was determined in the liver of Tg mice and WT littermates. (b) and (c) SFRP4 expression in the liver (B) and plasma (C) was determined by western blotting (β-actin was used as a loading control). A representative blot and data quantification from three independent experiments are shown. Data are expressed as the mean ± SEM, n = 6 for each group. *P < 0.05, **P < 0.01 vs. non-Tg

We successfully created Tg mice with liver-specific SFRP4 overexpression. The results showed that the hepatic levels of SFRP4 mRNA and protein were significantly higher in Tg compared to non-Tg mice (*P* < 0.05 or *P* < 0.01) (Figure 2(a,b)). The plasma level of SFRP4 protein was also compared by western blotting in the two groups of mice. Tg mice had significantly higher levels of plasma SFRP4 compared to non-Tg mice (*P* < 0.05) (Figure 2(c)), because of protein secretion by the liver.

### SFRP4 overexpression was associated with decreased size of visceral adipocytes

**Figure 3.**
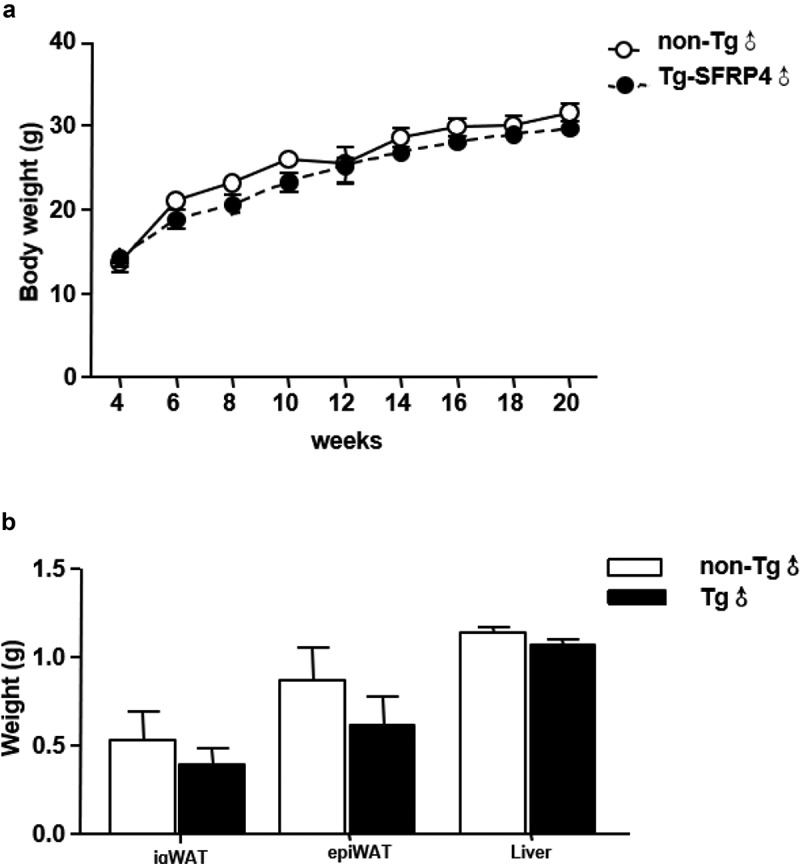
Body mass, fat mass, and liver weight in Tg mice. (a) Body weight changes in Tg mice and non-Tg littermates. (b) The ig WAT weight, epi WAT weight, and liver weight of non-Tg and Tg-SFRP4 mice. Data are expressed as the mean ± SEM, n = 8 for each group. **P < 0.01 vs. non-Tg

**Figure 4.**
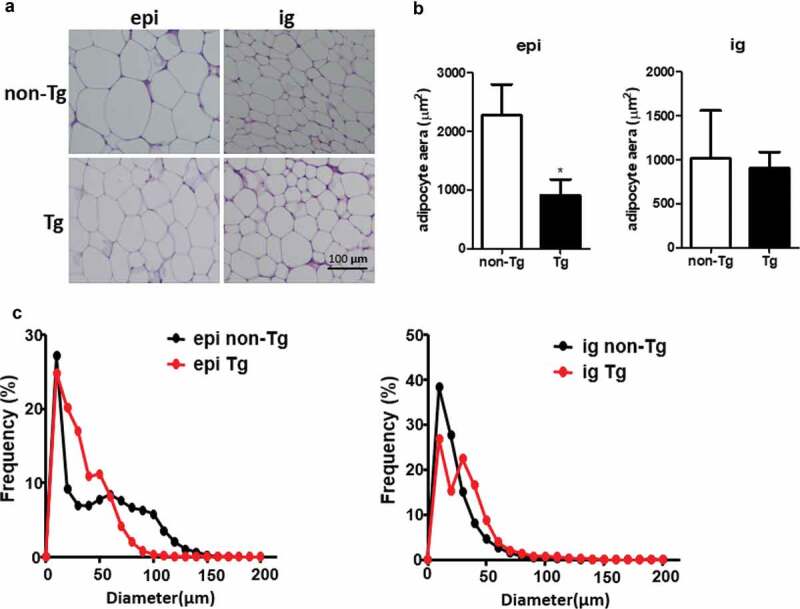
Adipocyte size was smaller in the epi WAT of Tg mice compared to WT littermates. (a) Representative image of adipocyte staining with haematoxylin & eosin. (b) The area of adipocytes was measured in Tg mice and non-Tg littermates. (c) A frequency distribution plot of cell diameters was calculated to determine the mean diameter of fat cells. Data are expressed as the mean ± SEM, n = 8 for each group. *P < 0.05 vs. non-Tg

Eight-week-old male Tg mice and control littermates were fed with chow diet for 12 weeks. There were no significant differences in body weight, fat mass and liver weight between Tg and non-Tg mice (Figure 3). H&E staining was used to explore the morphological characteristics of adipocytes in Tg and non-Tg mice. We found that the size of epi WAT adipocytes was smaller in Tg mice compared to control littermates. However, no significant differences were observed in the size of ig WAT adipocytes between the two groups (Figure 4).

### SFRP4 regulated the expression of genes involved in the differentiation of adipocytes from visceral adipose tissue

**Figure 5.**
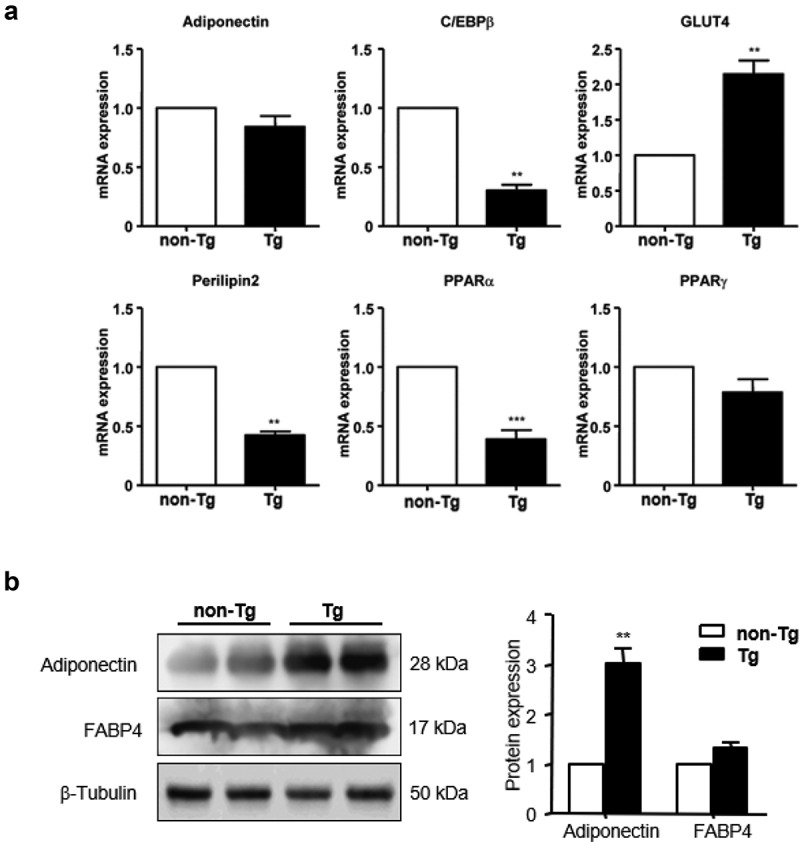
Effect of SFRP4 on gene expression in visceral adipose tissue. (a) The expression of adipogenic genes in epi WAT was determined by qPCR. (b) adipokine protein expression was determined by western blotting. Data are expressed as mean ± SEM, n = 6. **P < 0.01, ***P < 0.001 vs. non-Tg

The epi WAT of non-Tg and Tg male mice was isolated, total RNA and protein were extracted, and the expression of adipocytokines was analysed by qPCR and western blotting. The results showed that the mRNA expression of CCAAT/enhancer-binding protein-β (C/EBPβ), perilipin2, and peroxisome proliferator-activated receptor alpha (PPARα) was significantly decreased (*P* < 0.01), while that of GLUT4 was significantly increased (*P* < 0.01), in Tg mice compared to controls. No significant differences were observed in the expression of adiponectin and PPARγ (Figure 5(a)). Additionally, adiponectin protein expression was significantly higher in Tg compared to non-Tg mice, while FABP4 expression level was comparable in the two groups (Figure 5(b)).

### Effect of SFRP4 expression on subcutaneous adipose formation

**Figure 6.**
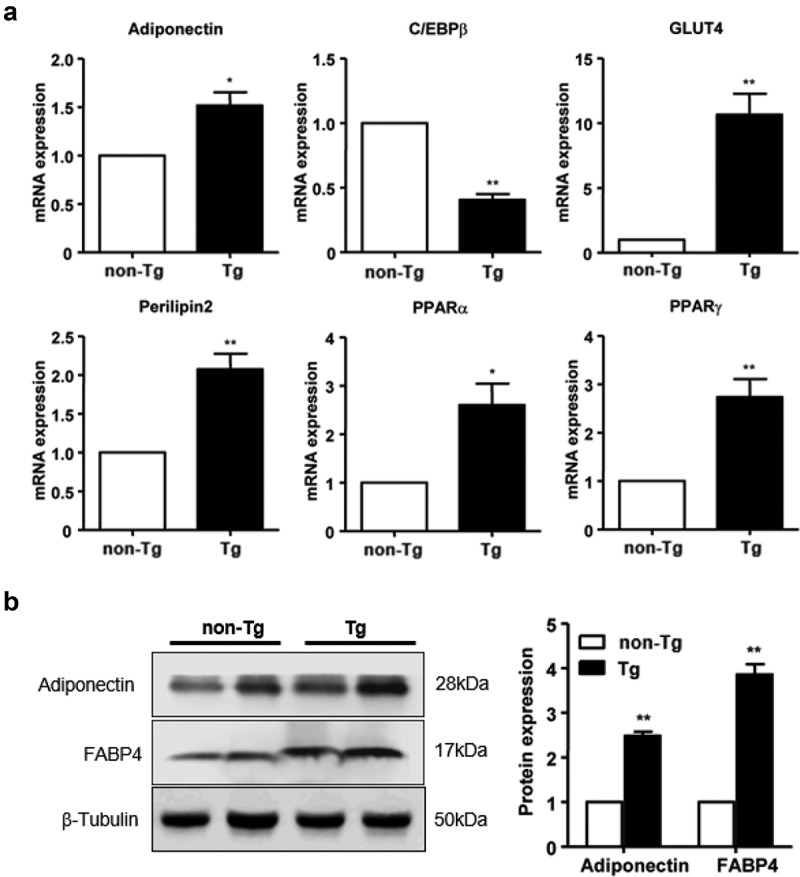
Effect of SFRP4 on gene expression in subcutaneous adipose tissue. (a) The expression of adipogenic genes in ig WAT was determined by qPCR. (b) Adipokine protein expression was determined by western blotting. Data are expressed as mean ± SEM, n = 6. *P < 0.05, **P < 0.01 vs. non-Tg

To determine the effect of *SFRP4* overexpression on subcutaneous adipose tissue, the ig WAT was collected from Tg mice and control littermates, and total RNA and protein were extracted. The mRNA expression of C/EBPβ was significantly lower in Tg mice compared to controls, while that of adiponectin, GLUT4, perilipin2, PPARα, and PPARγ was significantly increased in *SFRP4*-overexpressing mice (*P* < 0.05 or *P* < 0.01) (Figure 6(a)). Moreover, adiponectin and FABP4 protein levels were significantly higher in Tg mice than in control littermates (*P* < 0.01) (Figure 6(b)).

## Discussion

The main purpose of the current study was to investigate the impact of increased levels of circulating SFRP4 on adipose tissue deposition and glucose metabolism. In order to obtain an increase in the level of circulating SFRP4, we generated Tg mice overexpressing *SFRP4* under the human apoE/C-I hepatic control region, thus inducing preferential expression in the liver. In this preliminary study, we investigated the effects of systemic SFRP4 overexpression on adipose tissue.

SFRP4 was highly expressed in the adipose tissue of C57BL/6 and ob/ob mice, in line with previous studies reporting high SFRP4 expression in the human subcutaneous adipose tissue and in obese individuals [23,32]. However, we also found that SFRP4 did not contribute to fat deposition, since the Tg mice had no significant differences in body weight, and body composition (epi WAT, ig WAT, liver) compared with non-Tg mice, which was complemented by the related research in SFRP4 knock out mice [33].

We also found that *SFRP4* overexpression was associated with a reduction in the size of epididymal adipocytes, which may reflect substantial changes in metabolism [34,35]. Previous studies demonstrated that the size of adipocytes in the abdominal depot is a reliable predictor of type 2 diabetes [36–38]. Adipocyte hypertrophy contributes to insulin resistance, and *SFRP4* is overexpressed in type 2 diabetes mellitus [15,39,40]. It is worth mentioning that adiponectin expression was increased in the adipose tissue of Tg mice. Adiponectin, an adipokine, is the most abundant peptide secreted by adipocytes. Its level is decreased in obese individuals, and inversely correlated with insulin resistance and glucose intolerance [41,42]. *Adiponectin*-overexpressing mice show improved insulin sensitivity [43]. In contrast, *adiponectin*-deficient mice exhibit insulin resistance and glucose intolerance [44,45]. We found increased expression of GLUT4 in SFRP4 Tg mice. GLUT4 is an insulin-regulated glucose transporter, primarily expressed in the adipose tissue and striated muscle [46], which mediates glucose uptake, thus alleviating insulin resistance [47]. Our results suggested that *SFRP4* overexpression in mice might affect glucose metabolism. Of course, additional work is required to in-depth characterize the relationship between SFRP4 and insulin resistance.

Our previous *in vitro* experiments demonstrated that SFRP4 might play different roles in subcutaneous and visceral adipose tissue [25]. On the other hand, *in vivo* studies are the most suitable approach to investigate the function of SFRP4 in different types of adipose tissue. Due to its role in the storage and mobilization of triglycerides, the adipose tissue is a crucial regulator of energy homoeostasis. Its growth is characterized by an increase in adipocyte size, as well by the formation of new adipocytes from precursor cells (adipocyte proliferation) [48,49]. Major differences were previously observed in adipogenic potential between visceral and subcutaneous adipose tissue [50]. Moreover, adipocyte precursors from subcutaneous adipose tissue exert a protective effect against obesity-associated metabolic complications [51]. Furthermore, the accumulation of visceral adipose tissue is strongly associated with obesity-related complications, including type 2 diabetes and coronary artery disease [52]. Our results demonstrated that the expression patterns of genes related to adipocyte differentiation, including PPARγ, PPARα, perilipin 2, and FABP4, were distinct in visceral and subcutaneous adipose tissue, suggesting that SFRP4 may play different roles in the two tissues in mice. C/EBPs belong to a highly conserved family of leucine zinc finger proteins serving as transcription factors and include ɑ, β, and δ subtypes [53]. The three subtypes of C/EBPs contribute to the regulation of lipogenic gene expression, affect glucose uptake in adipocytes, and play important roles in different stages of adipocyte differentiation [53]. C/EBPβ is an early-stage regulator of adipocyte differentiation. It triggers lipid synthesis by inducing the expression of PPARγ and C/EBPα [54–56]. The development of adipose tissue is impaired in C/EBPβ knockout mice, and the efficiency of adipogenic differentiation is significantly reduced in the precursors of C/EBPβ [57]. Our results indicated that C/EBPβ mRNA levels were significantly decreased in both visceral and subcutaneous adipose tissues of Tg mice, compared to control mice. In the next study, the impact of SFRP4 on glucose metabolism and adipocyte differentiation will be explored in high-fat diet-induced obese Tg mice or, alternatively, by crossing these animals with ob/ob mice.

## Conclusion

Our study demonstrated that *SFRP4* overexpression in C57BL/6 N mice affected the size of epididymal adipocytes and the expression of adipokines. In particular, the expression of adiponectin and GLUT4 was found increased in Tg mice, suggesting a role of SFRP4 in glucose metabolism and adipocyte differentiation in mice.

## Abbreviations

C/EBP: CCAAT/enhancer-binding protein
epi WAT: epididymal white adipose tissue
FABP4: fatty acid binding protein-4
GLUT4: glucose transporter 4
ig WAT: inguinal white adipose tissue
PPAR: peroxisome proliferator activated receptor
SFRP4: secreted frizzled-related proteins
Tg: transgenic
WT: wild type
